# Development of a Mouse Model of Uremic Cardiomyopathy: Investigating the Impact of Chronic Kidney Disease on Cardiac Function and Signaling Pathway

**DOI:** 10.1096/fj.202500281R

**Published:** 2025-05-19

**Authors:** Julia Moellmann, Katja Glandien, Barbara M. Klinkhammer, Julia Wollenhaupt, Heidi Noels, Joachim Jankowski, Corinna Lebherz, Peter Boor, Michael Lehrke, Nikolaus Marx

**Affiliations:** ^1^ Department of Internal Medicine I University Hospital Aachen, RWTH Aachen University Aachen Germany; ^2^ Institute of Pathology University Hospital Aachen, RWTH Aachen University Aachen Germany; ^3^ Institute for Molecular Cardiovascular Research (IMCAR) RWTH Aachen University Aachen Germany; ^4^ Department of Nephrology RWTH Aachen University Aachen Germany

**Keywords:** 2,8‐dihydroxyadenine(DHA) nephropathy, cardiovascular disease, chronic kidney disease, uremic cardiomyopathy

## Abstract

Patients with chronic kidney disease are at an increased risk of developing heart failure, but the underlying mechanisms remain incompletely understood, at least in part because of the paucity of mouse models of uremic cardiomyopathy. In this study, we used two different experimental setups of 2,8‐dihydroxyadenine‐induced nephropathy in different mouse strains to develop a non‐invasive mouse model of uremic cardiomyopathy. Among the different models, only 129/Sv mice fed an adenine‐supplemented diet for 16 weeks showed typical features of uremic cardiomyopathy. Kidney damage was confirmed by histopathologic findings of diffuse fibrosis with collagen deposition, crystal formation, and uremia. The cardiac phenotype showed significantly increased myocardial fibrosis associated with impaired cardiac contractility under dobutamine‐induced stress conditions. This was associated with a significant activation of the mTOR pathway and downstream endoplasmic reticulum stress, increased apoptosis, and inflammation. Treatment of 129/Sv mice with an adenine‐supplemented diet for 16 weeks represents a model of uremic cardiomyopathy with increased myocardial fibrosis and impaired cardiac function, as well as a shift from cardioprotective to detrimental signaling, increased endoplasmic reticulum stress, and inflammation.

## Graphical abstract

Molecular changes in a mouse model of uremic cardiomyopathy. This model of 2,8‐DHA‐induced nephropathy in 129/Sv mice showed clinical features of uremic cardiomyopathy like reduced cardiac function and cardiac interstitial fibrosis and an unfavorable balance between protective and detrimental cardiac signaling.

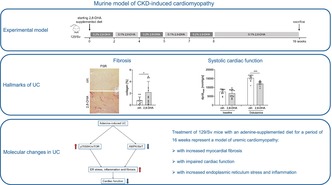

**Abbreviations**
ACC: Acetyl‐CoA‐Carboxylase
ADP: adenosine diphosphate
AMPK: AMP‐activated protein kinase
ATF4: activating transcription factor 4
BDH1: beta‐hydroxybutyrate dehydrogenase 1
BP: blood pressure
Casp3: caspase 3
CD36: cluster of differentiation 36
CHOP: C/EBP homology protein
CKD: chronic kidney disease
CVD: cardiovascular disease
DAPI: 4′,6‐Diamidin‐2‐phenylindol
DHA: dihydroxyadenine
dp/dtmax: maximum rate of intraventricular pressure rise
dp/dtmin: maximum rate of pressure change in the ventricle
eIF2α: eukaryotic initiation factor 2
GLUT4: glucose transporter type 4
IL‐1β: interleukin 1β
IL‐6: interleukin 6
KDEL: Grp78 and Grp94
mTOR: mechanistic target of rapamycin
OCR: oxygen consumption rate
P/M: pyruvate/malate
p70S6K: p70 S6 kinase
PAS: periodic acid‐schiff
PSR: picro sirius red
SCOT: succinyl‐CoA:3‐oxoacid‐CoA transferase
SirT: sirtuine
UC: uremic cardiomyopathy

## 1 Introduction

Patients with chronic kidney disease (CKD) are at increased risk of developing cardiovascular disease (CVD), including sudden cardiac death, arrhythmias, coronary artery disease, and heart failure, with an approximately 75% increased risk in patients with CKD stage 4–5 [1, 2]. The term uremic cardiomyopathy (UC) describes a CKD‐induced cardiac damage leading to cardiac dysfunction with the characteristic features of left ventricular hypertrophy and myocardial fibrosis [3]. UC is attributed to CKD‐dependent fluid retention, activation of the renin‐angiotensin system, oxidative stress, or uremic toxins leading to direct cardiac injury with activation of inflammatory pathways [4].

However, CKD is an independent and additive risk factor beyond the classical drivers of CVD, including age, smoking, diabetes, dyslipidemia, and hypertension. Nevertheless, the underlying mechanisms of UC remain incompletely understood [5]. This lack of knowledge is at least partly due to the lack of reproducible, especially non‐surgical mouse models of UC, which have been difficult to develop and have yielded partially contradictory results. Therefore, we performed a protocol to induce CKD in different mouse strains and to evaluate the effect on cardiac changes with the aim of establishing a mouse model of UC with the typical characteristics of cardiac hypertrophy and myocardial fibrosis as seen in CKD patients. We focused on the method of 2,8‐dihydroxyadenine (DHA)‐induced CKD model as a more easily applicable alternative to surgery‐induced CKD.

## 2 Methods

### 2.1 Animal Studies

All experiments were approved by the government of North Rhine‐Westphalia approval number: 81–02.04.2017.A447 (model 1) and 81–02.04.2017.A425 (model 2) (Germany). These studies were performed in seven‐week‐old male mice obtained from Janvier Labs and maintained on a 12‐h day/night cycle with ad libitum food and water at the animal facility of the University Hospital RWTH Aachen University. Three different strains were used: C57BL/6 J and N (C57BL/6JRj and C57BL/6NRj) and 129/Sv (129S2/SvPasOrlRj) mice. Animal experiments were started after a 1 week acclimation period to our facility following the importation of the mice. CKD was induced by feeding the mice an adenine‐supplemented diet (2,8‐dihydroxyadenine (DHA) nephropathy) (ssniff Spezialdiäten GmbH, Soest, Germany) in two different mouse studies as described in detail below. At the end of the experiment, overnight‐fasted mice were anesthetized with ketamine (100 mg/kg) and xylazine (10 mg/kg), and Temgesic (0.1 mg/kg) was administered for analgesia. Hemodynamics were measured with a Millar catheter (Millar Instruments, Texas, USA) after the catheter was advanced via the right carotid artery through the aortic valve into the left ventricle. Basal measurements were followed by i.p. injection of dobutamine (5 mg/kg body weight) as a cardiac stressor. Signals were continuously recorded and analyzed using iox (emka Technologies, Paris, France) [6]. Blood samples were collected, mice were sacrificed by cervical dislocation, and before tissue samples were collected and used immediately or snap frozen in liquid nitrogen and stored at −80°C until further analysis, the heart and vasculature were rinsed with ice‐cold PBS. Serum urea was determined at the animal facility of the University Hospital RWTH Aachen (Germany).

**FIGURE 1:**
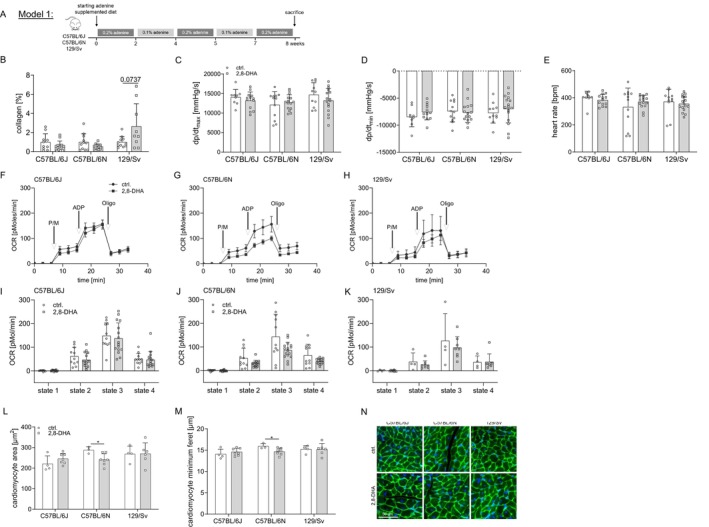
CKD‐induced increase in cardiac fibrosis only in 129/Sv mice. We treated different mouse strains (C57BL/6J, C57BL/6N and 129/Sv mice) with an adenine‐supplemented or control diet for a period of 8 weeks (A) (alternating time periods: 2 weeks 0.2%, 2 weeks 0.1%, 1 week 0.2%, 2 weeks 0.1% and 1 week 0.2% adenine). We found uremia increased cardiac fibrosis only in 129/Sv mice (B) (C57BL/6J: ctrl.: *n* = 10 and 2,8‐DHA: *n* = 13; C57BL/6N: ctrl.: *n* = 11 and 2,8‐DHA: *n* = 13; 129/Sv: ctrl.: *n* = 11 and 2,8‐DHA: *n* = 9). Examining cardiac function, we found no difference in left ventricular functionality in response to uremic conditions in any of the strains studied (C–E) (C57BL/6J: ctrl.: *n* = 9 and 2,8‐DHA: *n* = 13; C57BL/6N: ctrl.: *n* = 12 and 2,8‐DHA: *n* = 15 and 129/Sv: ctrl.: *n* = 11 and 2,8‐DHA: *n* = 16). Furthermore, no difference in myocardial mitochondrial function was observed in any of the strains analyzed (F–K) (F/I: C57BL/6J: ctrl.: *n* = 11 and 2,8‐DHA: *n* = 16; G/J: C57BL/6N: ctrl.: *n* = 11 and 2,8‐DHA: *n* = 16 and H/K: 129/Sv: ctrl.: *n* = 4 and 2,8‐DHA: *n* = 8). No difference in cardiomyocyte hypertrophy was found in C57BL/6J or 129/Sv mice, whereas we unexpectedly found that uremic conditions reduced cardiomyocyte size in C57BL/6N mice (L–N) (C57BL/6J: ctrl.: *n* = 4 and 2,8‐DHA: *n* = 7; C57BL/6N: ctrl.: *n* = 3 and 2,8‐DHA: *n* = 7 and 129/Sv: ctrl.: *n* = 4 and 2,8‐DHA: *n* = 6). Results are expressed as mean ± SD and analyzed by two‐tailed *t*‐test ((parametric data; with Welch's correction in case of non‐equal SDs if mentioned) or Mann–Whitney's test (non‐parametric data)) (B–E (B: Welch's correction for C57BL/6N and 129/Sv; E: Welch's correction for C57BL/6N) and I–M (I: Mann–Whitney's test for state 1/4; J: Mann–Whitney's test for state 2 and Welch's correction for state 3/4; K: Welch's correction for state 3 and Mann–Whitney's test for state 4) or two‐way repeated measures ANOVA with matching values and Sidak's post‐test (F–H); *p < 0.05, **p < 0.01 and ***p < 0.001.

Experimental design 1: We investigated the consequences of uremia in different mouse strains. Therefore, CKD was simultaneously induced in C57BL/6J, C57BL/6N and 129/Sv by feeding them an adenine‐supplemented diet (2,8‐DHA nephropathy) for 8 weeks according to the following schedule (Figure 1A): 2 weeks with 0.2% of adenine, 2 weeks with 0.1% of adenine, 1 week with 0.2% of adenine, 2 weeks with 0.1% of adenine, and finally 1 week with 0.2% of adenine.

Systolic and diastolic blood pressure (BP) measurements were obtained by the non‐invasive tail‐cuff method using the CODA system (Kent Scientific Corporation, USA). Non‐anesthetized mice were placed in animal holders, and the tails were placed in a specially designed cuff connected to the system. Blood flow was interrupted by automatic cuff inflation, and flow pulsation was subsequently detected by a built‐in sensor during slow air expiration. To reduce stress‐related errors in the measurements, the mice were trained at least 2 days prior to the experiment to acclimate them to the experimental procedure [7].

**FIGURE 2:**
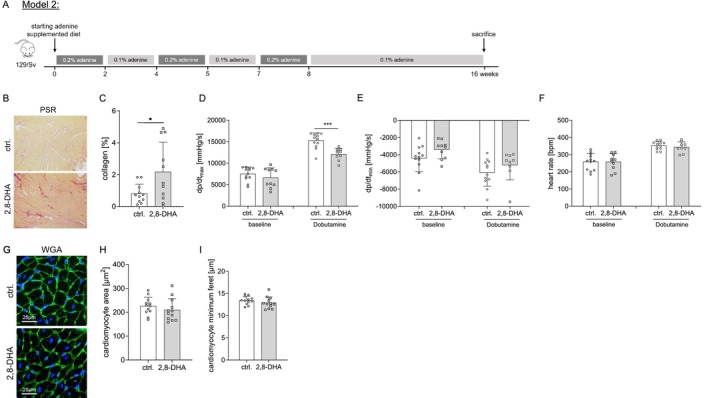
CKD‐dependent morphological and functional cardiac changes in 129/Sv mice. In this model, we treated 129/Sv mice with an adenine‐supplemented diet to induce 2,8‐dihydroxyadenine (DHA) crystal formation for a total of 16 weeks (A) (alternating time periods: 2 weeks 0.2%, 2 weeks 0.1%, 1 week 0.2%, 2 weeks 0.1%, 1 week 0.2% and 8 weeks 0.1% adenine; *n* = 12 ctrl. and *n* = 12 2,8‐DHA mice). Examining the cardiac phenotype, we found a significant increase in myocardial fibrosis (B/C) (*n* = 11 ctrl. and *n* = 11 2,8‐DHA mice) in association with a significant impairment of left ventricular contractility as an indicator of systolic function under dobutamine stress conditions (D), whereas cardiac relaxation, heart rate and cardiomyocyte hypertrophy were not affected (E–I) (D–F: Baseline: *n* = 12 ctrl. and *n* = 12 2,8‐DHA and dobutamine: *n* = 10 ctrl. and *n* = 9 2,8‐DHA mice and H/I: *n* = 12 ctrl. and *n* = 12 2,8‐DHA mice). Results are expressed as mean ± SD and analyzed by two‐tailed t‐test ((parametric data; with Welch's correction in case of non‐equal SDs) (C/H/I) C: Welch's correction)) or mixed effects analysis with matched values and Sidak's post‐test (D–F); *p < 0.05, **p < 0.01 and ***p < 0.001.

Experimental design 2: 129/Sv mice were fed an adenine‐supplemented or control diet for 16 weeks to induce long‐term CKD (schematic illustration Figure 2A): CKD induction during the first eight weeks was performed as in experimental design 1 (initiation phase). The concentration was then reduced to 0.1% of adenine for further 8 weeks (maintenance phase) to achieve a prolonged period of CKD for a total of 16 weeks.

### 2.2 Western Blot Analysis

Heart tissue samples were homogenized in lysis buffer (0.25 M sucrose, 2.2 mM Na_2_‐EDTA, 10 mM Tris) using a TissueLyser LT (Qiagen, Hilden, Germany) and supplemented with phosphatase inhibitors (PhosSTOP, #4906837001) and protease inhibitors (cOmplete, #11697498001) (both Roche, Basel, Switzerland). 40 μg of protein lysate was separated on a 4%–15% gradient gel before transfer to a nitrocellulose membrane. All antibodies were purchased from Cell Signaling (Massachusetts, USA) and used at a 1:1000 dilution unless otherwise noted: ACC (#3676), p‐ACC(Ser79) (#11818), AMPKα (#2603), p‐AMPKα(Thr172) (#4188), ATF4 (#11815), CASP3 (#14220), CHOP (#2895), eIF2α (#5324), p‐eIF2α(Ser51) (#3398), GLUT4 (#2213), IL‐1β (#12703), mTOR (#2983), p‐mTOR(Ser2448) (#5536), p‐mTOR(Ser2481) (#2974), p70S6K (#2708), p‐p70S6K(Thr389) (#9206), SirT3 (#5490), CD36 (#NB400; Novus Biologicals), BDH1 (#MA5‐15594; 1:500; ThermoFisher), SCOT (#ab105320; 1:500; Abcam), and KDEL (#ADI‐SPA‐827; 1:1000; Enzo). For Ponceau S staining, nitrocellulose membranes were incubated in Ponceau S solution (0.1% (w/v) Ponceau S (#P3504; Merck, Darmstadt, Germany) in 5% (v/v) acetic acid) and destained with deionized water. HRP‐conjugated anti‐rabbit or anti‐mouse IgG antibodies were used as secondary antibodies. Western blots were detected using a Chemi Doc MP Imaging System (BioRad, California, USA) and analyzed using Image Lab 5.0 software (BioRad) [6].

### 2.3 Histological Analysis

Heart tissues were fixed in 4% paraformaldehyde overnight and embedded in paraffin. Hearts were sectioned from the top to the apex, and 4 μm sections were taken after the mitral valve. Interstitial collagen was visualized by Picro‐Sirius Red (PSR) staining and analyzed using Image‐Pro Plus 7.0 software (Media Cybernetics) [6].

For analysis of cardiomyocyte size and area, tissue sections were rehydrated and heat‐induced antigen retrieval was performed in citrate buffer. Slides were incubated with fluorescein‐conjugated wheat germ agglutinin ((WGA), 1:100 (Vector Laboratories)) and counterstained with 4′,6‐diamidin‐2‐phenylindol (DAPI). Slides were digitized using an Aperio Versa200 whole slide scanner (Leica Biosystems). 150–200 cross sections of cardiomyocytes per mouse were measured and analyzed using ImageJ (NIH) [6].

Kidney tissues were fixed in methyl Carnoy's solution overnight and embedded in paraffin. For general morphological assessment, 1 μm sections were stained with periodic acid‐Schiff (PAS) stain. Tubulointerstitial injury (including tubular cell flattening and dilation, atrophy, vacuolization, inflammation, fibrosis) was scored as follows: 0 = 0%–1% of the area with signs of injury, 1 = 2%–25%, 2 = 26%–50%, 3 = 51%–75%, and 4 = 76%–100%. H&E staining was used to quantify kidney 2,8‐DHA crystals. For this purpose, images of the kidney cortex were captured with 20x objectives under polarized light, and the positive bright crystal area was analyzed using ImageJ (NIH). To quantify kidney fibrosis, immunostaining for collagen type III was performed using goat anti‐type III collagen (Southern Biotech) as the primary antibody and biotinylated rabbit anti‐goat IgG (Vector Laboratories) as the secondary antibody. Visualization was performed by using AB complex signal enhancement followed by incubation with 3,3′‐diaminobenzidine. Slides were digitized using an Aperio AT2 whole slide scanner (Leica Biosystems), and the positively stained area in the cortex and medulla was quantified using Image Scope (Leica Biosystems) [8].

### 2.4 Mitochondrial Analysis

Heart or kidney tissue samples were collected in isolation buffer (IB) (IB: 225 mM mannitol, 75 mM sucrose, 2 mM HEPES, 1 mM EGTA; pH = 7.4). Lysis was performed in IB supplemented with 4 mg/mL BSA (#A7030) and 1.6 mg/mL proteinase (#P8038) (both Merck, Darmstadt, Germany) with metal beads at 25 Hz in a TissueLyser LT (Qiagen, Hilden, Germany) for 3 min. This step was repeated after the addition of the same volume of lysis buffer for another 3 min at 25 Hz. The suspensions were centrifuged at 400 g for 5 min at 4°C to collect the supernatant. After a second centrifugation of the supernatant at 7700 g for 10 min at 4°C, the pellet was resuspended in mitochondrial suspension buffer (MSB) (MSB: 225 mM mannitol, 75 mM sucrose, 2 mM HEPES, pH = 7.4). After two further washing steps, including centrifugation at 7700 g for 5 min each at 4°C, the isolated mitochondria were stored in MSB and the oxygen consumption rate (OCR) was determined from 40 μg mitochondria in respiratory buffer (137 mM KCl, 2 mM KH_2_PO_4_, 2.5 mM MgCl_2_, 20 mM HEPES, 0.5 mM EGTA; pH = 7.2) using a Seahorse analyzer (Agilent Technologies, California, USA). We measured mitochondrial function using the following protocol: first, mitochondrial OCR was measured under baseline conditions ‐ only freshly isolated mitochondria without substrate in respiratory buffer (state 1). Then, state 2 respiration was induced by substrate supplementation (pyruvate/malate) (P/M; 5 mM), i.e., low ADP level and a high ATP/ADP ratio. In addition, adenosine diphosphate (ADP; 1 mM) (#A2754) was added to detect state 3 respiration, which implies a high level of (extramitochondrial) ADP and a low ADP/ATP ratio. Finally, the F_1_F_0_ ATP synthase inhibitor oligomycin A (1.2 μM) (#75351) (both Merck, Darmstadt, Germany) was added (state 4). Each cycle was measured in three replicates of 30 s mixing and 2 min measuring procedure [8].

### 2.5 Blood Sample Analysis

The concentration of interleukin‐6 (IL‐6) in plasma was determined by an enzyme‐linked immunosorbent assay (ELISA) according to the manufacturer's protocol (R & D Systems).

### 2.6 Statistical Analysis

Data are presented as mean ± SD. All statistical data analyses, including normal distribution tests and graph preparation, were performed using GraphPad Prism (version 10). Statistics included two‐way repeated measures ANOVA with matched values and Sidak's post‐test and, in the presence of missing values, mixed‐effects analysis with Sidak's post‐test for multiple comparisons of matched values (values from one animal at different time points) with normal distribution; student's t‐test (unpaired, two‐tailed) for two‐group comparisons with normal distribution and without time variables, with additional Welsh's correction of non‐equal standard deviations; Mann–Whitney test for two‐group comparisons without normal distribution, as detailed indicated in the figure legends. Outliers were excluded using the Grubbs' test in GraphPad. P values are defined as follows: *p < 0.05, **p < 0.01, ***p < 0.001, and ****p < 0.0001.

## 3 Results

### 3.1 Cardiac Effects of 2,8‐DHA‐Induced CKD in Different Mouse Strains

We treated C57BL/6J, C57BL/6N, and 129/Sv mice with an adenine‐supplemented diet to induce 2,8‐DHA crystal formation or with a control diet for 8 weeks (Figure 1A). We observed the expected induction of uremia (Figure S1A), kidney crystal formation (Figure S1B/C) with tubulointerstitial injury (Figure S1D/E) and kidney collagen deposition (Figure S1F–H) in all strains. Furthermore, kidney mitochondrial function was impaired in all strains (Figure S1: C57BL/6J (I/L), C57BL/6N (J/M) and 129/Sv (K/N)), while systolic blood pressure was increased in C57BL/6J and C57BL/6N but not in 129/Sv mice (Figure S1O–Q). With respect to characteristic features of UC, only 129/Sv mice developed an increase in cardiac fibrosis after 8 weeks of adenine diet (Figure 1B). There was no difference in cardiac functionality in response to uremic conditions in any of the investigated strains studied (Figure 1C–E). Furthermore, no difference in myocardial mitochondrial function was observed in any of the strains studied (Figure 1: C57BL/6J (F/I), C57BL/6N (G/J) and 129/Sv (H/K)). In addition, no changes in cardiomyocyte hypertrophy were observed in any of the strains (Figure 1L–N).

### 3.2 CKD‐Dependent Morphological and Functional Cardiac Changes in 129/Sv Mice

Because of the exclusive induction of cardiac fibrosis by uremic conditions in 129/Sv mice, we decided to perform further analyses in this mouse strain only. Therefore, we treated 129/Sv mice with an adenine‐supplemented diet for 16 weeks (Figure 2A). This resulted in the expected kidney damage with collagen deposition (Figure S2A–C), crystal formation (Figure S2D/E) and uremia (Figure S2F). In addition, we measured plasma levels of IL‐6, a widely used biomarker of inflammation and disease progression in experimental models. IL‐6 levels increased only slightly throughout the experimental period, with no differences observed between CKD mice and controls (Figure S2G).

**FIGURE 3:**
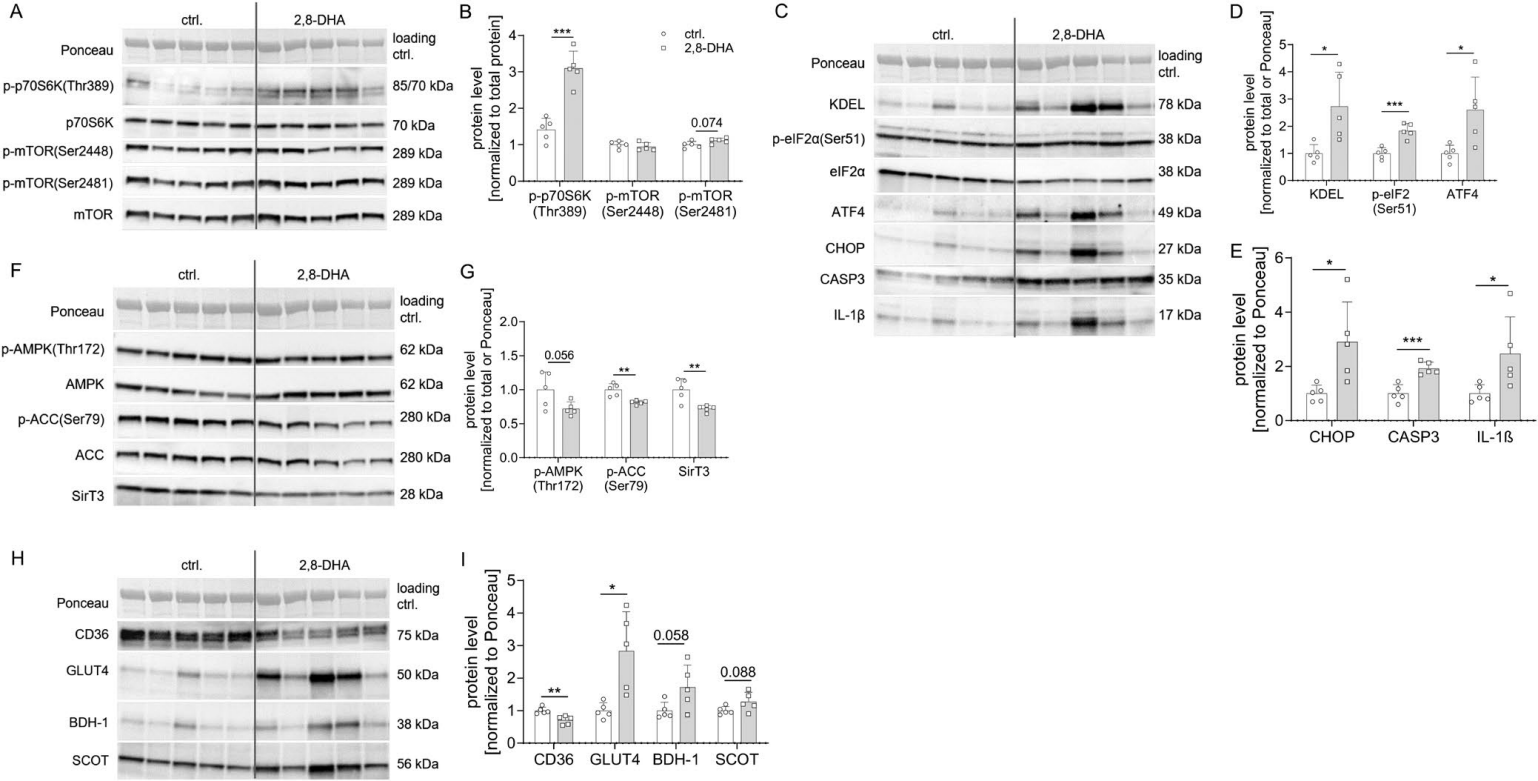
CKD‐dependent mTOR activation in 129/Sv mice. Investigating the metabolic and inflammatory signature underlying this cardiac phenotype, we found significant activation of the mTOR pathway (as indicated by its downstream target p70S6K‐phosphorylation at Thr389) (A/B) along with downstream endoplasmic reticulum stress (as indicated by eIF2α‐phosphorylation at Ser51, ATF4 and CHOP), increased apoptosis (via caspase 3 expression) and inflammation (via IL‐1β expression) (C–E). Furthermore, we observed decreased AMPK signaling and Sirt3 expression (F/G), and suppression of the fatty acid transporter CD36 expression, while insulin dependent glucose transporter GLUT4 expression was increased (H/I). No difference was observed in the expression of the ketone catabolizing enzymes BDH‐1 and SCOT (H/I) (all A/C/F/H: ctrl.: *n* = 5 and 2,8‐DHA: *n* = 5). Results are expressed as mean ± SD and analyzed by two‐tailed *t*‐test ((parametric data; with Welch's correction for unequal SDs when mentioned)) (B/D/E/G/I (D: Welch's correction for KDEL and ATF4; E: Welch's correction for CHOP and IL‐1β; G: Welch's correction for SirT3; I: Welch's correction for GLUT4)); *p < 0.05, **p < 0.01 and ***p < 0.001.

Investigating the cardiac phenotype, we observed a significant increase in myocardial interstitial fibrosis (Figure 2B/C) associated with a significant impairment in cardiac contractility as an indicator of systolic function under dobutamine stress conditions (Figure 2D). In contrast, cardiac relaxation, heart rate, and cardiac hypertrophy were not affected (Figure 2E–I). When investigating the metabolic and inflammatory features underlying this cardiac phenotype, we observed significant activation of the mTOR pathway as evidenced by the phosphorylation of its downstream target p70S6K at Thr389 (Figure 3A/B). In addition, there was notable downstream endoplasmic reticulum stress, as indicated by the phosphorylation of eIF2α at Ser51 and the presence of ATF4 and CHOP. We also detected increased apoptosis (as evidenced by increased caspase 3 expression) and increased inflammation as evidenced by increased IL‐1β expression (Figure 3C,D/E). The 129/Sv strain showed altered signaling patterns in the heart with a decrease in protective pathways and an increase in detrimental pathways as observed by decreased AMPK signaling and Sirt3 expression (Figure 3F/G) and suppressed expression of the fatty acid transporter CD36, whereas the expression of the insulin‐dependent glucose transporter GLUT4 was increased (Figure 3H/I). No difference was observed in the expression of the ketone catabolizing enzymes BDH‐1 and SCOT (Figure 3H/I). Furthermore, we did not find differences in the expression of lipogenic enzymes (*Fasn* and *Srebf1*; data not shown), the mitochondrial fatty acid transporter *Cpt1* (data not shown), mitochondrial transcription factors (*Ppargc1a*, *Tfam* and *Nrf1*; data not shown) or markers of mitochondrial fission and fusion (data not shown), nor in mitochondrial respiration (data not shown).

In conclusion, this model of 2,8‐DHA‐induced nephropathy in 129/Sv mice induced clinical features of uremic cardiomyopathy (reduced cardiac function, cardiac interstitial fibrosis; Table 1) as well as an unfavorable balance between protective and detrimental cardiac signaling.

**TABLE 1:** Features of uremic cardiomyopathy.

	Strain	Treatment	Time [weeks]	Kidney			Heart			
				Fibrosis	Serum urea	Mitochondrial function	Cardiac contractility	Fibrosis	Hypertrophy	Mitochondrial function
Model 1	C57BL/6J	Adenine	8	Yes	↑	↓	/	/	/	/
	C57BL/6N			Yes	↑	↓	/	/	↓	↓
	129/Sv			Yes	↑	↓	/	/	/	/
	FVB (N)			Yes	↑	/	/	/	/	/
Model 2	S129/Sv	Adenine	16	Yes	↑	↓	↓	↑	/	/

Abbreviations: /, no change; –, not analyzed.

## 4 Discussion

In this study, we established a mouse model of UC by simultaneously comparing several mouse strains with different durations of adenine treatment. Our approach led to the development of a non‐surgical mouse model of UC. In 129/Sv mice fed an adenine‐supplemented diet for 16 weeks, we found increased cardiac fibrosis and impaired cardiac function. This was associated with increased mTOR signaling, downstream endoplasmic reticulum stress, and induction of inflammatory and pro‐fibrotic signaling.

Mouse models of UC have been difficult to establish, with partially conflicting and strain‐dependent results, as summarized in a recent meta‐analysis. To complicate matters, these studies have used different protocols for CKD induction and measures of cardiac changes, making it difficult to rigorously compare UC in different mouse strains [9]. In this study, we used C57BL/6 mice as the strain with the greatest availability of genetic mouse models. In our hands, CKD did not induce UC in either C57BL/6J or C57BL/6N mice, as indicated by the absence of cardiac fibrosis, in contrast to some, but not all, of the studies summarized in the afore mentioned meta‐analysis. In addition, we observed an increase in systolic blood pressure only in C57BL/6J and C57BL/6N mice, but not in 129/Sv mice. We are still unable to explain this. The observed difference with previous reports may be due to the method used to induce CKD by crystal‐induced nephropathy in our study and subtotal 5/6 nephrectomy in most of the other studies. Furthermore, the surgical method of CKD induction has not been reproducible even up to 16 weeks of CKD with respect to the development of cardiac fibrosis or other markers of UC in C57BL/6 mice [7, 9]. The absence of typical features of UC was evident in our study in both C57BL/6N and C57BL/6J mice, with C57BL/6J previously identified as less susceptible to mitochondrial reactive oxygen species production due to the genetic absence of a functional mitochondrial nicotinamide nucleotide transhydrogenase (NNT) [10, 11]. In contrast to the C57BL/6 strains, we showed that after 8 weeks of CKD induction, 129/Sv mice exhibited at least cardiac fibrosis and altered cardiac signaling, suggesting an unfavorable shift from protective to detrimental pathways. Thus, by providing a direct comparison of different mouse strains with identical experimental protocols of CKD induction performed simultaneously to exclude potential time‐dependent factors, our study indicates that C57BL/6 mice are at least partially protected from UC in direct comparison to 129/Sv mice after 8 weeks.

Therefore, we prolonged the treatment with adenine supplementation specifically in 129/Sv mice up to 16 weeks. This significantly increased cardiac interstitial fibrosis (2.9‐fold) and caused reduced cardiac contractility under dobutamine stress conditions. These observations are consistent with studies using subtotal nephrectomy in 129/Sv mice, which also reported CKD‐dependent cardiac fibrosis and hypertrophy. However, to our knowledge, there is no mouse model of UC available that is independent of surgery [9, 12]. In comparison, two previous studies of 2,8‐DHA‐induced CKD (for 8 weeks in C57BL/6) reported only either cardiac fibrosis or reduced cardiac function, but not both features of UC [13, 14]. A third study examining the heart after 20 weeks of 0.15% adenine diet in C57BL/6 reported reduced ejection fraction and cardiac hypertrophy along with increased cardiac fibrosis, although the latter remained < 5% elevated compared to controls [15]. Furthermore, neither in 129/Sv nor in any other mouse strain studied to date has a 2,8‐DHA‐induced CKD protocol been reported that can simultaneously induce myocardial interstitial fibrosis, cardiac inflammation, and impaired cardiac function [9, 12], as observed in our regimen of adenine treatment in the 129/Sv strain. The relevant reasons for the strain‐specific differences in UC susceptibility are currently unknown. 129/Sv mice have two renin genes, but we did not observe a higher basal or CKD‐induced blood pressure increase in this mouse strain compared to the others, making this an unlikely explanation for the cardiac phenotype. Furthermore, the 129/Sv strain has been reported to be more susceptible to kidney injury, fibrosis, and inflammation after subtotal nephrectomy [16], but this was not observed in our study during the parallel induction of 2,8‐DHA‐induced CKD in terms of serum urea, tubulointerstitial injury, or kidney fibrosis. We also did not observe any differences in plasma IL‐6 levels, a typical biomarker of inflammation and disease progression in experimental models, in our CKD mouse model. Most likely, a combination of genetic differences underlies our observed differences in cardiac presentation upon CKD induction in 129/Sv compared to the other strains. Genome sequence comparison of inbred mouse strains revealed that the 129/Sv strain has > 1500 protein‐coding genes and > 2400 protein‐coding transcripts with at least one amino acid truncation, expansion, or change with a minimum defined “severity impact” in terms of predicted effect on protein function compared to the C57BL/6J strain [17]. Our model now facilitates the investigation of the underlying mechanisms of CKD‐dependent cardiovascular changes and the testing of new therapeutic interventions in a preclinical mouse model. To better understand the molecular cardiac changes, we investigated metabolic and pro‐fibrotic signaling pathways. CKD reduced cardiac AMPK and SirT3 signaling both cardioprotective [18, 19] in 129/Sv mice as a possible mechanism of mTOR activation leading to downstream endoplasmic reticulum stress. This occurred in conjunction with increased cardiac expression of the glucose transporter GLUT4 and suppression of the fatty acid transporter CD36, suggesting a possible switch in cardiac substrate metabolism to favor glucose over fatty acid use. Since increased fatty acid oxidation has recently been shown to be cardioprotective against heart failure [20], such molecular alterations might induce susceptibility to adverse cardiac changes. Nevertheless, we did not detect differences in cardiac mitochondrial function or metabolic intermediates (data not shown), suggesting that metabolic alterations and energy deficit are an unlikely mechanism for cardiac damage and fibrosis in 129/Sv mice.

As a relevant limitation, the presented results of our study were not performed in direct comparison with the CKD model induced by 5/6 nephrectomy analyzed only in male mice. Since the aim of this project was to establish a model of uremic cardiomyopathy in mice and to comply with the 3R requirements (3R: Replacement, Reduction and Refinement), initial experiments (model 1) were performed with a small number of animals and an evaluation of only eight and 16 weeks of CKD induction. Future research is warranted to evaluate the effect of our 2,8‐DHA CKD model also in females and on other parameters of cardiac function. In addition, the present results of our study remain descriptive and we are not able to identify the main causative factors for CKD‐mediated UC, which demonstrates the need for further investigation.

Given the high clinical relevance of CKD‐associated CVD, the aim of this study was to investigate the cardiac function and the pathophysiological features of uremic cardiomyopathy in different mouse strains with CKD. We established a non‐surgical mouse model of uremic cardiomyopathy using 129/Sv mice fed an adenine‐supplemented diet for 16 weeks. We observed increased cardiac fibrosis and impaired cardiac function, closely mimicking key pathological features known from human disease.

As mentioned above, additional studies are needed to establish the causality between the observed pathway changes and the cardiac phenotype. Furthermore, we are currently unable to describe the exact mechanism leading to mTOR activation as a likely cause of the pro‐fibrotic phenotype in our study. However, the establishment of a mouse model of uremic cardiomyopathy using 2,8‐DHA‐induced nephropathy in 129/Sv mice now allows further analysis to gain a better understanding of the molecular mechanisms leading to the development of cardiac changes in CKD.

## Author Contributions

J.M. contributed to the experimental design, execution, and analysis of the study and drafted the manuscript. K.G. contributed to the experimental execution of the study. B.M.K. contributed to the histological analysis of the kidney and heart. B.M.K. and P.B. contributed to the critical discussion of the results and to the revision/editing of the manuscript. J.W., H.N., J.J., and C.L. participated in the critical discussion of the results and drafting of the manuscript. N.M. and M.L. contributed to the experimental design and drafting of the manuscript, are the guarantors of this work and as such had full access to all the data in the study and take responsibility for the integrity of the data and the accuracy of the data analysis.

## Disclosure

M.L. received grants and personal fees from Boehringer Ingelheim, MSD, Novo Nordisk, and personal fees from Amgen, Sanofi, Astra Zeneca, Bayer, Lilly, Daiichi Sankyo, Novartis. N.M., H.N., and J.J. are founding shareholders of AMICARE Development GmbH. J.M., K.G., B.M.K., J.W., H.N., J.J., C.L., and P.B. have nothing to disclose.

## Conflicts of Interest

The authors declare no conflicts of interest.

## Supporting information


**Figure S1.** Different strains—C57BL/6J, C57BL/6N and 129/Sv mice—developed the expected uremia and kidney injury. To study only the consequences of uremia in different mouse strains, the following three strains were used: C57BL/6J, C57BL/6N and 129/Sv mice and fed an adenine‐supplemented or control diet. This resulted in the expected induction of uremia (A), kidney crystal formation (B/C) with tubulointerstitial injury (D/E) and collagen deposition (F/G/H) in all strains (A/C/E/G/H: all *n* = 4 ctrl. and *n* = 8 2,8‐DHA per strain; except 129/Sv: *n* = 12 ctrl. and *n* = 16 2,8‐DHA). At the final time point, kidney mitochondrial function was impaired in C57BL/6J, C57BL/6N and 129/Sv mice (I–K) (I/L: C57BL/6J: ctrl.: *n* = 11 and 2,8‐DHA: *n* = 18; J/M: C57BL/6N: ctrl.: *n* = 12 and 2,8‐DHA: *n* = 18 and K/N: 129/Sv: ctrl.: *n* = 4 and 2,8‐DHA: *n* = 8), whereas systolic blood pressure was increased in C57BL/6J, C57BL/6N but not in 129/Sv mice (O–Q) (C57BL/6J: ctrl.: *n* = 4 and 2,8‐DHA: *n* = 8; C57BL/6N: ctrl.: *n* = 4 and 2,8‐DHA: *n* = 8 and 129/Sv: ctrl.: *n* = 4 and 2,8‐DHA: *n* = 8 [expected 5 weeks 2,8‐DHA: *n* = 7]). Results are expressed as mean ± SD and analyzed by two‐tailed *t*‐test ([parametric data; with Welch’s correction for unequal SDs] or Mann–Whitney test [non‐parametric data]) (A/C/E/G/H/L–N) (A/C: Welch’s correction for C57BL/6J and C57BL/6N; C: Mann–Whitney test for 129/Sv; E: Mann–Whitney test for C57BL/6J and Welch’s correction for C57BL/6N; M: Welch’s correction for state 1/3; N: Mann–Whitney test for state 1), two‐way repeated measures ANOVA with matched values and Sidak’s multiple comparison test (I–K, O/P) or mixed‐effects analysis with matched values and Sidak’s post‐test (Q); *p < 0.05, **p < 0.01, ***p < 0.001 and ****p < 0.0001.
**Figure S2.** CKD‐dependent morphological renal and systemic changes in 129/Sv mice. Feeding an adenine‐supplemented diet for 16 weeks leads to the expected kidney damage with collagen deposition (A–C) (*n* = 12 ctrl. and *n* = 12 2,8‐DHA mice), crystal formation (D/E) (*n* = 12 ctrl. and *n* = 11 2,8‐DHA mice) and uremia (F) (*n* = 10 ctrl. and n = 11 2,8‐DHA mice). Unexpectedly, we found no significant differences in plasma IL‐6 levels in our model (G) (basal: *n* = 14 ctrl. and *n* = 12 2,8‐DHA mice and final: *n* = 10 ctrl. and n = 10 2,8‐DHA mice). Results are expressed as mean ± SD and analyzed by two‐tailed *t*‐test (parametric data; with Welch’s correction for unequal SDs) or Mann–Whitney test (nonparametric data) (B/C/E/F/G (B/C/F: Welch’s correction; E: Mann–Whitney test)); *p < 0.05, **p < 0.01 and ***p < 0.001.

## Data Availability

The data that support the findings of this study are available on request from the corresponding author. The data are not publicly available due to privacy or ethical restrictions.
