# Supplementary figures and images for: Development of a Mouse Model of Uremic Cardiomyopathy: Investigating the Impact of Chronic Kidney Disease on Cardiac Function and Signaling Pathway
Source: FASEB J. 2025 May 19;39(10):e70639. doi: 10.1096/fj.202500281R (PMC12086955; doi:10.1096/fj.202500281R)

## Slide 1
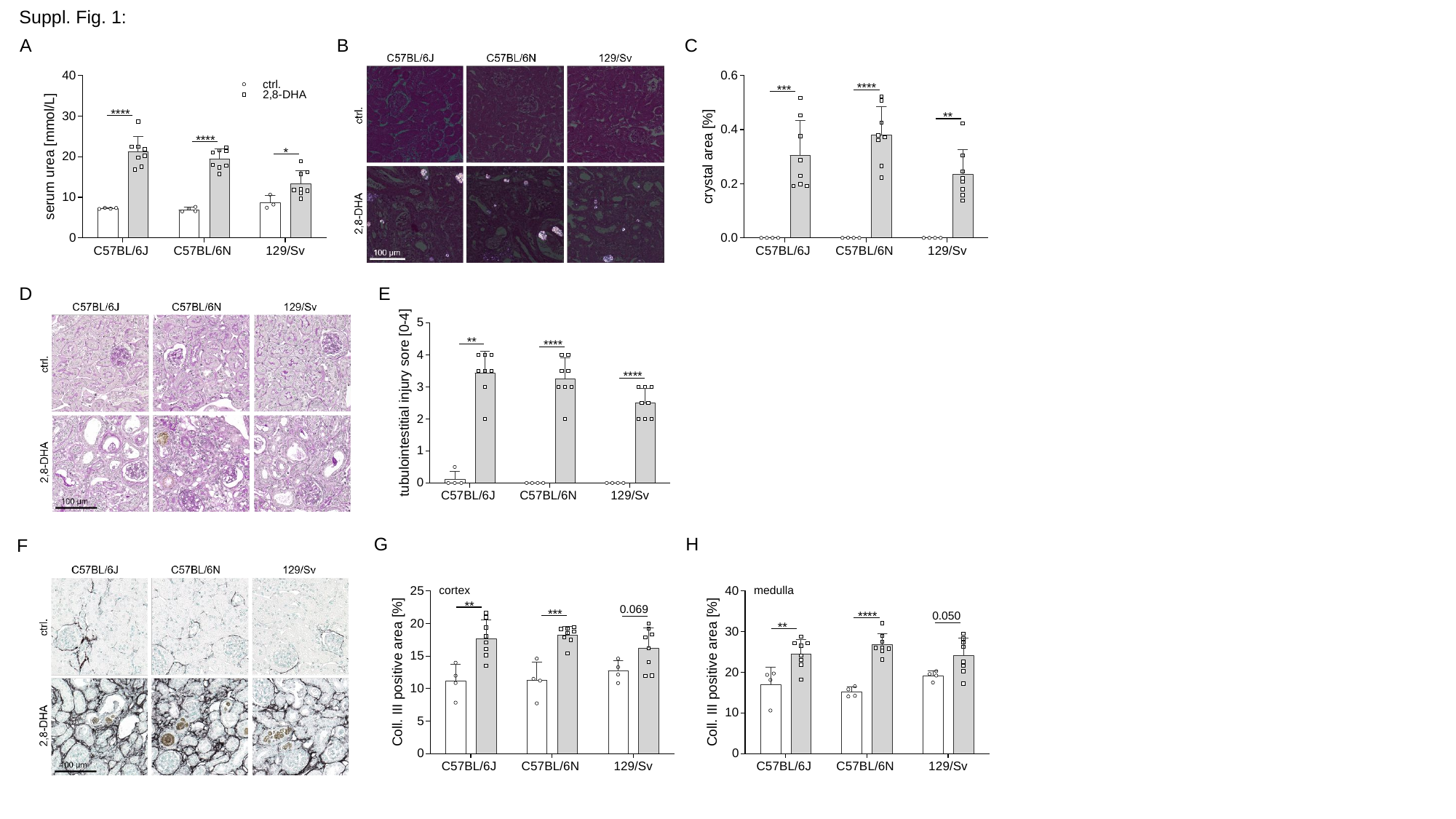

Suppl. Fig. 1:
A
B
C
D
E
H
G
F

## Slide 2
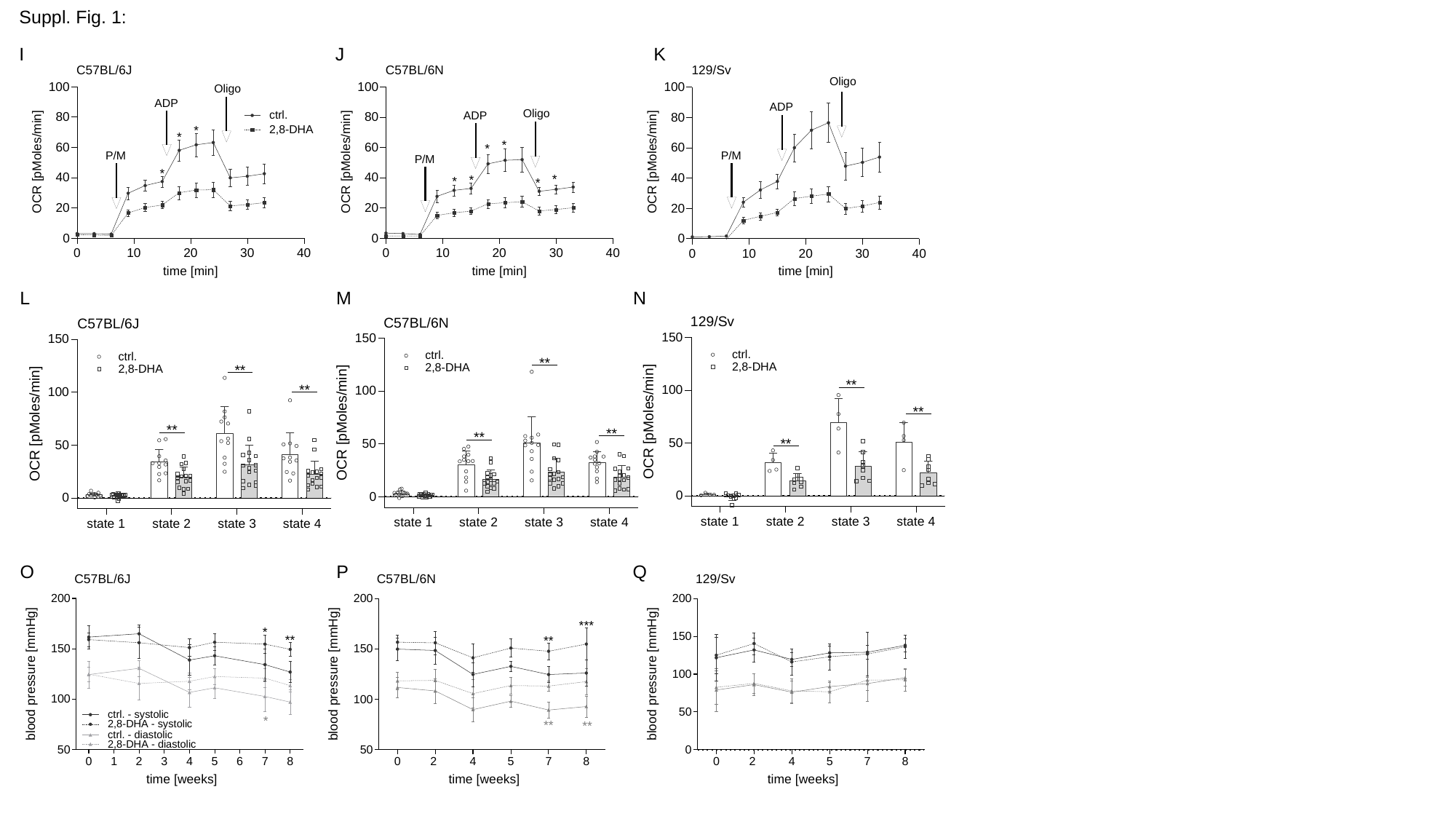

Suppl. Fig. 1:
I
J
K
L
M
N
O
P
Q

## Slide 3
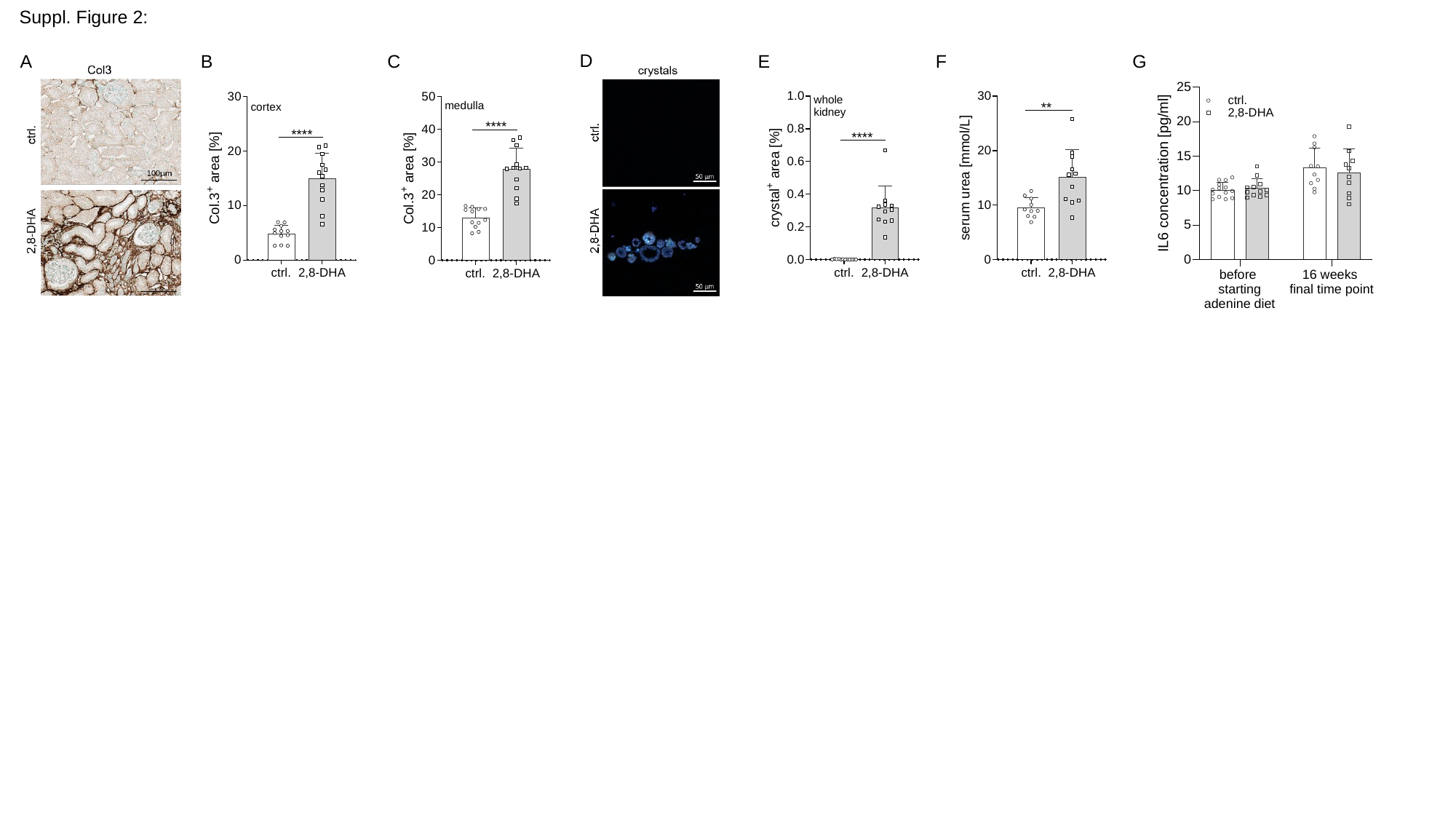

Suppl. Figure 2:
D
A
B
C
E
F
G

Supplement: Supplementary file 1 — Figure S1. Different strains—C57BL/6J, C57BL/6N and 129/Sv mice—developed the expected uremia and kidney injury. To study only the consequences of uremia in different mouse strains, the following three strains were used: C57BL/6J, C57BL/6N and 129/Sv mice and fed an adenine‐supplemented or control diet. This resulted in the expected induction of uremia (A), kidney crystal formation (B/C) with tubulointerstitial injury (D/E) and collagen deposition (F/G/H) in all strains (A/C/E/G/H: all n = 4 ctrl. and n = 8 2,8‐DHA per strain; except 129/Sv: n = 12 ctrl. and n = 16 2,8‐DHA). At the final time point, kidney mitochondrial function was impaired in C57BL/6J, C57BL/6N and 129/Sv mice (I–K) (I/L: C57BL/6J: ctrl.: n = 11 and 2,8‐DHA: n = 18; J/M: C57BL/6N: ctrl.: n = 12 and 2,8‐DHA: n = 18 and K/N: 129/Sv: ctrl.: n = 4 and 2,8‐DHA: n = 8), whereas systolic blood pressure was increased in C57BL/6J, C57BL/6N but not in 129/Sv mice (O–Q) (C57BL/6J: ctrl.: n = 4 and 2,8‐DHA: n = 8; C57BL/6N: ctrl.: n = 4 and 2,8‐DHA: n = 8 and 129/Sv: ctrl.: n = 4 and 2,8‐DHA: n = 8 [expected 5 weeks 2,8‐DHA: n = 7]). Results are expressed as mean ± SD and analyzed by two‐tailed t‐test ([parametric data; with Welch’s correction for unequal SDs] or Mann–Whitney test [non‐parametric data]) (A/C/E/G/H/L–N) (A/C: Welch’s correction for C57BL/6J and C57BL/6N; C: Mann–Whitney test for 129/Sv; E: Mann–Whitney test for C57BL/6J and Welch’s correction for C57BL/6N; M: Welch’s correction for state 1/3; N: Mann–Whitney test for state 1), two‐way repeated measures ANOVA with matched values and Sidak’s multiple comparison test (I–K, O/P) or mixed‐effects analysis with matched values and Sidak’s post‐test (Q); *p < 0.05, **p < 0.01, ***p < 0.001 and ****p < 0.0001. Figure S2. CKD‐dependent morphological renal and systemic changes in 129/Sv mice. Feeding an adenine‐supplemented diet for 16 weeks leads to the expected kidney damage with collagen deposition (A–C) (n = 12 ctrl. and n = 12 2,8‐DHA mice), crystal [file FSB2-39-e70639-s001.pptx]
